# Occupational exposure to noise in relation to pregnancy-related hypertensive disorders and diabetes

**DOI:** 10.5271/sjweh.3913

**Published:** 2020-12-16

**Authors:** Claudia Tyemi Lissåker, Per Gustavsson, Maria Albin, Petter Ljungman, Theo Bodin, Mattias Sjöström, Jenny Selander

**Affiliations:** 1 Unit of Occupational Medicine, Institute of Environmental Medicine, Karolinska Institutet, Stockholm, Sweden; 2 Centre for Occupational and Environmental Medicine, Region Stockholm, Sweden; 3 Unit of Environmental Epidemiology, Institute of Environmental Medicine, Karolinska Institutet, Stockholm, Sweden; 4 Department of Cardiology, Danderyd Hospital, Stockholm, Sweden

**Keywords:** employment, gestational hypertension, gestational diabetes, hypertension, JEM, job exposure matrix, noise exposure, occupational health, preeclampsia, work-related factor

## Abstract

**Objectives:**

Exposure to environmental noise has been associated with an increased risk of cardiovascular diseases and diabetes, but evidence for occupational noise is limited and conflicting, especially related to pregnancy outcomes. This study aimed to evaluate the association of occupational noise exposure with hypertensive disorders of pregnancy (HDP) and gestational diabetes.

**Methods:**

Our population-based cohort study utilized data on 1 109 516 singletons born to working mothers in Sweden between 1994–2014 from the Medical Birth Register and the Longitudinal Integration Database for Health Insurance and Labor Market Studies. Noise exposure came from a job exposure matrix (JEM) in five categories <70, 70–74, 75–80, 80–85, >85 dB(A). Relative risks (RR), adjusted for confounders and other job exposures, were calculated by modified Poisson regressions for the full sample and a subsample of first-time mothers reporting full-time work.

**Results:**

Exposure to 80–85 dB(A) of noise was associated with an increased risk of all HDP [RR 1.12, 95% confidence interval (CI) 1.05–1.18] and preeclampsia alone (RR 1.14, 95% CI 1.07–1.22) in the full sample. Results were similar for first-pregnancy, full-time workers. Exposure to >85 dB(A) of noise was also associated with an increased risk of gestational diabetes (RR 1.57, 95% CI 1.10–2.24) in the analysis restricted to first-time mothers working full-time.

**Conclusion:**

In this study, exposure to noise was associated with an increased risk for HDP and gestational diabetes, particularly in first-time mothers who work full-time. Further research is needed to confirm findings and identify the role of hearing protection on this association so prevention policies can be implemented.

Hypertensive disorders of pregnancy (HDP) – which include gestational hypertension, preeclampsia, and eclampsia – and gestational diabetes mellitus, are some of the most common conditions during pregnancy, affecting between 1–15% of pregnancies throughout the world (1, 2). HDP can lead to adverse birth outcomes, such as preterm placenta separation, preterm birth, and small for gestational age (3). Preeclampsia and eclampsia, in particular, are leading causes of maternal mortality, accounting for approximately 13% of maternal deaths in developed countries (4). Among those who survive, women who were diagnosed with HDP are more likely to develop a cardiovascular disease in the future (5). Likewise, gestational diabetes can lead to adverse outcomes both during and after pregnancy. For instance, pregnancies in women with gestational diabetes are more likely to include complications such as large-for-gestational age and neonatal hypoglycemia. Both women and their offspring are then later at an increased risk for type 2 diabetes (2).

Approximately one quarter of all hypertension cases during pregnancy will progress to preeclampsia; however, predicting who progresses has proven difficult (6). The causes of HDP in general are not well understood. Similarly, the etiology of gestational diabetes mellitus, has not been fully elucidated. Both disorders resolve after birth, share many of the same risk factors – such as increased age, obesity, and insulin resistance (3, 7) – and are associated with an increased risk for cardiovascular comorbidities in the long-term. These characteristics give rise to the theory that perhaps these disorders, and in particular preeclampsia, are a result of one of two patterns: (i) women without predisposing conditions who suffer from stresses additional to the normal adaptation to pregnancy, or (ii) women with weaker vasculature and/or predisposing condition in which the adaptation to pregnancy pushes them into the disordered state (8). These patterns, of course, could happen in a continuum, in which both external stressors and predisposing conditions interact and potentially lead to HDP or gestational diabetes.

Several studies have linked environmental noise exposure to cardiovascular disease (9–11) and diabetes (12) in the general population. However, a recent review by the World Health Organization concluded that there is not enough evidence of sufficient quality to make firm conclusions regarding the effects of noise on pregnancy (13). The workplace is an important and understudied source of noise exposure outside of the home and, significantly, offers the potential for prevention strategies. In fact, in Sweden, approximately 20% of women report being exposed for at least one quarter of their working day to noise so loud they cannot carry a conversation in a normal tone of voice (14). Globally this burden is even higher as developing countries move to more industrial economies (15). Despite the widespread nature of occupational sources of noise, little research has been done to investigate its impact on pregnancy outcomes. Studies focusing on gestational hypertension report mixed results, with only a small number of cases, and often relying on self-reported data (16–20). To our knowledge, no current study has investigated the impact of occupational noise on gestational diabetes.

The aim of this study is to explore whether noise exposure during pregnancy is associated with HDP, looking specifically at women who develop preeclampsia alone, as well as gestational diabetes, using a large, register-based, prospective dataset, adjusting for other occupational exposures and potential cofounders.

## Methods

### Data collection

A prospective cohort was formed based on information on each pregnancy from the Swedish Medical Birth Register for all births between 1994–2014. In this register, covering approximately 99% of all pregnancies (21), data are recorded from prenatal care unit visits occurring around week 10 of pregnancy until the birth of the child. Data include background characteristics collected in early pregnancy (such as date of birth, occupation, smoking status, weight, height, and nationality) as well as information on previous pregnancies, parity, and any diagnoses received during pregnancy. For this study, we only included women whose pregnancies resulted in single births and who reported working full- or part-time at the time of the first appointment at the prenatal care unit occurring on or around gestational week 10. Additionally, women who did not report an occupation were not included.

This register was merged with the Longitudinal Integration Database for Health Insurance and Labor Market Studies (Swedish acronym LISA) utilizing the unique personal identification number assigned to all individuals living in Sweden in order to obtain information on socioeconomic characteristics, such as mother’s highest education at the time of delivery and marital status. LISA has complete coverage of all individuals aged 16 and older residing in Sweden.

### Noise exposure

To examine noise exposure, we used a job exposure matrix (JEM) linked to the cohort data using occupational codes. Occupational information provided at the prenatal care unit was recorded as free text, which was then coded into the ISCO-88-based standard for Swedish occupational classification 96 (SSYK96) coding system by an occupational hygienist. The coding details are presented elsewhere (22). SSYK96 is provided in a four-digit level, with each digit, from left to right, providing greater detail regarding the occupation.

The noise exposure job matrix includes information on annual average 8-hour occupational noise level in decibel [dB(A)] in five-year intervals from 1970–2014 for 321 occupations. Noise levels were derived from measurement reports from occupational health services and clinics as well as large companies throughout Sweden and were specified in five categories: <70, 70–74, 75–80, 80–85, >85 dB(A) (23). A previous version of this matrix has been shown to be valid (23). We linked these levels to the maternal data based on the occupational code and birth year.

Some occupations did not have enough detail to be given a 4-digit SSYK96 code and were given a 2- or 3- digit code; thus, they could not be matched directly with the JEM job groups. For these cases, imputation was done in one of two ways. First, if all occupations within this 2- or 3-digit job groups had the same noise category, that category was imputed for the missing observations. For instance, all occupations within the ‘manager of small companies and entities’ job group had the lowest noise level; therefore, women who were coded as belonging to this overall job group were also given the same level. Second, if the noise levels varied within the 2- or 3-digit job group, an occupational hygienist with expertise on noise exposure and blinded to the diagnoses of individuals within each group was consulted, and he then assigned the most likely noise level for those observations.

### Outcome

For each mother–child pair included in our study, we extracted diagnoses coded at the end of each pregnancy reported in the Medical Birth Register. These were coded using the International Classification of Diseases, ninth and tenth revisions (ICD-9 and ICD-10). For HDP, ICD-9 codes 642, 642D, 642E, 642F, 642G, and 642H and ICD-10 codes O11, O13, O14.0, O14.1, O14.2, O14.9, O15.0, O15.1, O15.2, and O15.9 were used. For preeclampsia, ICD-9 codes 642E, 642F, and 642H and ICD-10 codes O11.9, O14.0, O14.1, O14.2, O14.9, O15.0, O15.1, O15.2, and O15.9 were used. Finally, for gestational diabetes, ICD-9 codes 648A and 648W and ICD-10 codes O24.4 and O24.9 were used.

### Potential confounders

Several potential confounders were considered. From the Medical Birth Register we obtained maternal age at delivery (<25, 25–30, 30–35, or ≥35 years), smoking status at week 10 (non-smokers, smokers), educational level (some high school or less, high school graduate, some university or higher), marital status (not in a registered partnership or living alone, married or in a registered partnership), family situation (living with the father, living alone or another arrangement), body mass index (BMI) calculated using height and weight [underweight (<18.5 kg/m^2^), normal weight (18.5–25 kg/m^2^), overweight (25–30 kg/m^2^), obese (≥30 kg/m^2^)], country of birth (Sweden, Europe excluding Sweden, and rest of the world), parity (first pregnancy, ≥1 pregnancy), and employment status (full-time, part-time). Occupational exposures considered were whole body vibrations, any particle exposure, physically strenuous work, job strain, and exposure to low temperatures, which were obtained from various JEM and matched to each woman based on occupational code and year, if applicable.

Whole body vibration was categorized as 0–0.1, 0.1–0.3, 0.3–0.5, and >0.5 m/s^2^. The occupations that did not have enough detail (coded on a 2- or 3-digit SSYK96) were those in which all sub-groups (coded on a 4-digit SSYK96) were not exposed to vibrations; therefore, all were assigned a value of 0. We also considered occupational exposure to particles by including a JEM based on the Finnish Information System on Occupational Exposure JEM (24) and adapted to Swedish conditions, which included estimates for 24 agents: asbestos, silica, lead, iron, nickel, chromium, concrete/stone, other inorganic dust, wood, animal, paper, textile, flour, oil mist, cooking fumes, other organic dust, asphalt, diesel, benzo(a)pyrene, polycyclic aromatic hydrocarbons, other combustion fumes, and welding fumes. An occupation that was exposed to any of these agents was classified as exposed to particles. The physical workload, psychosocial stress, and temperature JEM have continuous measures based on questionnaires. For these JEM, when SSYK96 codes did not have enough detail, we calculated an average weighted according to the population distribution of Swedish women working in the sub-groups each occupational group. In other words, if a sub-group had a greater proportion of women compared to other sub-groups, their exposure was given more weight in the average calculation. A total of 15 894 (1.4%) women were given imputed values. Physical load was used as an average of several physical exposures (bending, heavy lifting, stooping, working in a twisted position, rapid breathing, working with hands above shoulder level, physically strenuous work, and repetitive work), divided into quartiles. We used the decision authority and demand domains of the psychosocial stress JEM to create the job strain variable. A job exposed to above the mean value of either domain was considered to be exposed to high levels, and below the mean to low levels. Values for cold temperatures were divided into tertiles.

### Data analyses

For all analyses, we included women who had a single birth and reported being employed (full-time or part-time). Covariate adjustment followed a two-step procedure. First, we selected separate sets of confounders for each analytical model based on known risk factors for HDP and gestational diabetes (3, 7), placement on a causal diagram (25–27), and impact on the association between exposure and each outcome using a 5% cutoff. Then, in a second step, to ensure consistency and ease of interpretation between outcomes, if a variable was selected into one model, it was entered into the final model for all other outcomes. All covariates previously mentioned were considered for inclusion, and ultimately, confounders added to the final model were: maternal age at delivery, smoking status, educational level, country of birth, parity, employment status, any particle exposure, physical load, job strain, and exposure to low temperatures.

To explore multicollinearity between the exposure and confounders, we included them into a linear regression model and calculated the variance inflation index (VIF). VIF values were well <5 for all categories of all covariates, indicating that, statistically, there were no major issues with multicollinearity.

Because different births from the same mother could not be assumed to be independent, relative risks (RR) were estimated using a modified Poisson regression for correlated binary data (28). Crude and adjusted models were created for each outcome (HDP, preeclampsia, gestational diabetes), with the adjusted model including complete cases only. We repeated the analysis with a subsample of women with a first-time pregnancy who reported full-time employment to investigate the association for those who are most likely exposed to the category of noise assigned to their job code. Women, with previous pregnancy complications may work less or change their work tasks during a subsequent pregnancy and those who work part-time will be exposed to less noise than those who work full-time. The same modified Poisson regression was utilized to estimate RR; however, no correlation adjustment was necessary as each woman only contributed with one observation. Further sensitivity analyses were done on the full sample by investigating age interactions. All statistical analyses were performed using SAS version 9.4 (SAS Institute, Cary, NC, USA).

This study was conducted with approval from the regional ethical review board in Stockholm, Sweden.

## Results

A total of 1 109 516 mother-child pairs were included in the study. The characteristics of the cohort are described in table 1. The majority (63%) of women were exposed to the <70 dBA in their workplace; 19% were exposed to 70–75 dBA, 5% to 75–79 dBA, 12% to 80–84 dBA, and <1% were exposed to levels >85 dBA. Compared to those who were exposed to the lowest noise category, women who were exposed to the highest category of noise (>85 dBA) were younger, more often smokers, more often had lower levels of education, and were born in European countries other than Sweden. These women were also more often employed full-time, and were concomitantly more often exposed to particles, high physical load, job strain, and cold temperatures. Table 2 lists the ten most common occupations within each exposure category.

**Table 1:** Baseline maternal characteristics and occupational exposure to noise during pregnancy.

	Total	Occupational noise exposure				

		<70 dB(A) (N=699 783)	70–74 dB(A) (N=211 500)	75–79 dB(A) (N=59 993)	80–85 dB(A) (N=128 586)	≥85 dB(A) (N=9654)
				
		N (%)	N (%)	N (%)	N (%)	N (%)
Age at delivery (years)						
<25	121 892 (11.0)	64 190 (9.2)	27 711 (13.1)	10 421 (17.3)	19 517 (14.2)	1681 (17.4)
5–30	363 024 (32.7)	213 677 (30.5)	75 165 (35.5)	20 977 (35.0)	53 075 (38.5)	3826 (39.6)
30–35	404 751 (36.5)	270 713 (38.7)	70 975 (33.6)	18 769 (31.3)	44 169 (32.0)	2857 (29.6)
≥35	219 849 (19.8)	151 203 (21.6)	37 649 (17.8)	9826 (16.4)	21 086 (15.3)	1290 (13.4)
Smoking status						
Non-smoker	1 007 024 (90.8)	641 041 (91.6)	189 627 (89.7)	51 766 (86.3)	116 608 (90.7)	7982 (82.7)
Smoker	86 996 (7.8)	48 860 (7.0)	18 873 (8.9)	7452 (12.4)	10 282 (8.0)	1529 (15.8)
Missing	15 496 (1.4)	9882 (1.4)	3000 (1.4)	775 (1.3)	1696 (1.3)	143 (1.5)
Educational level						
Some high school or less	280 825 (25.3)	154 165 (22.0)	60 392 (28.5)	23 127 (38.5)	38 346 (29.8)	4795 (49.7)
High school graduate	308 940 (27.8)	200 852 (28.7)	54 890 (26.0)	20 628 (34.5)	28 576 (22.2)	3940 (40.8)
Some university or higher	514 586 (46.4)	342 083 (48.9)	94 665 (44.8)	15 782 (26.3)	61 178 (47.6)	878 (9.1)
Missing	5165 (0.5)	2683 (0.4)	1553 (0.7)	402 (0.7)	486 (0.4)	41 (0.4)
Country of birth						
Sweden	983 489 (88.6)	625 711 (89.4)	183 688 (86.9)	50 919 (84.9)	114 705 (89.2)	8466 (87.7)
Europe (excluding Sweden)	64 226 (5.8)	37 938 (5.4)	14 628 (6.9)	4371 (7.3)	6511 (5.1)	778 (8.1)
Rest of the world	60 743 (5.5)	35 487 (5.1)	12 956 (6.1)	4635 (7.7)	7259 (5.6)	406 (4.2)
Missing	1058 (0.1)	647 (0.1)	228 (0.1)	65 (0.1)	111 (0.1)	4 (0)
Parity						
First pregnancy	513 560 (46.3)	326 622 (46.7)	97 946 (46.3)	29 404 (49.0)	55 228 (43.0)	4360 (45.2)
≥1 pregnancy	595 956 (53.7)	373 161 (53.3)	113 554 (53.7)	30 589 (51.0)	73 358 (57.0)	5294 (54.8)
Employment status						
Full-time	725 494 (65.4)	464 959 (66.4)	125 574 (59.4)	42 624 (71.0)	84 057 (65.4)	8280 (85.8)
Part-time	384 022 (34.6)	234 824 (33.6)	85 926 (40.6)	17 369 (29.0)	44 529 (34.6)	1374 (14.2)
Any particle exposure						
Yes	139 786 (12.6)	16 490 (2.4)	55 608 (26.3)	37 492 (62.5)	21 448 (16.7)	8748 (90.6)
No	969 730 (87.4)	683 293 (97.6)	155 892 (73.7)	22 501 (37.5)	107 138 (83.3)	906 (9.4)
Physical load (quartiles)						
^st^	282 512 (25.5)	268 088 (38.3)	9817 (4.6)	4607 (7.7)	0 (0)	0 (0)
2^nd^	268 262 (24.2)	185 728 (26.5)	75 059 (35.5)	7377 (12.3)	98 (0.1)	0 (0)
^rd^	316 680 (28.5)	125 016 (17.9)	80 376 (38.0)	6254 (10.4)	104 903 (81.6)	131 (1.4)
4^th^	242 062 (21.8)	120 951 (17.3)	46 248 (21.9)	41 755 (69.6)	23 585 (18.3)	9523 (98.6)
Job strain (decision authority/demand)						
High/high	345 178 (31.1)	319 288 (45.6)	6858 (3.2)	16 506 (27.5)	2526 (2.0)	0 (0)
High/low	214 311 (19.3)	137 738 (19.7)	55 333 (26.2)	15 763 (26.3)	3807 (3.0)	1670 (17.3)
Low/high	210 792 (19.0)	51 041 (7.3)	85 649 (40.5)	16 351 (27.2)	57 123 (44.4)	628 (6.5)
Low/low	339 235 (30.6)	191 716 (27.4)	63 660 (30.1)	11 373 (19.0)	65 130 (50.7)	7356 (76.2)
Low temperature (tertiles)						
1^st^	372 828 (33.6)	303 583 (43.4)	64 464 (30.5)	3619 (6.0)	357 (0.3)	805 (8.3)
2^nd^	365 823 (33.0)	291 342 (41.6)	53 769 (25.4)	17 059 (28.5)	3360 (2.6)	293 (3.0)
3^rd^	370 865 (33.4)	104 858 (15.0)	93 267 (44.1)	39 315 (65.5)	124 869 (97.1)	8556 (88.6)

**Table 2:** Ten most common occupations within each noise category. Due to changes in classification over the years, one occupation can appear within more than one category.

Occupation (%)				

<70 dB(A)	70–75 dB(A)	75–80 dB(A)	80–85 dB(A)	≥85 dB(A)
Assistant nurses (12.9)	Midwives (27.4)	Chefs (18.8)	Daycare teachers & workers (44.4)	Machine operators, wood products (66.2)
Health & personal assistants (10.5)	Retail workers (26.3)	Head waiters, waiters & bartenders (18.4)	Child caretakers (35.1)	Butchers, meat cutters (6.3)
University teachers (8.6)	Hotel & office cleaners (12.8)	Automotive assembly workers (13.4)	Restaurant & kitchen staff (12.1)	Carpenters (5.5)
Other office personnel (5.7)	Warehouse assistants (3.4)	Youth recreational leaders (10.1)	Machine tool operator (2.1)	Welder & flame cutter (4.4)
Accountants & accounting assistants (4.6)	Primary school teachers (2.5)	Upper secondary school teacher in common subjects (6.4)	Composers, classical musicians & singers (2.0)	Machine operators, textile industry (2.3)
B2B sales (3.7)	Teacher in practical & arts subjects (2.5)	Dentists (4.5)	Production worker, meat & fish industry (1.4)	Lumber mill workers (2.2)
Hairdressers & beauticians (2.7)	Dental assistants (2.1)	Tailors & seamstresses (3.2)	Other production workers (0.6)	Production workers, paper industry (1.9)
Managers for smaller companies & units (2.5)	Police officers (2.1)	Production workers, bakery factory workers (2.8)	Production workers, bakery industry (0.4)	Production worker, meat & fish industry (1.4)
Secretaries, medical secretaries (2.4)	Healthcare attendants (2.0)	Farmers, fruit & berries (2.6)	Automotive assembly worker (0.4)	Blacksmiths (1.2)
Social workers (2.4)	Postal workers (1.9)	Bakers & confectioners (2.1)	Production worker, plastics industry (0.2)	Production workers, breweries (1.1)

Results from the regression analyses for the full sample, which includes both full- and part-time workers, as well as first and subsequent pregnancies, are found in table 3. After adjusting for all confounders, exposure to the second highest category of noise (80–85 dBA) was associated with an increased risk of HDP of pregnancy (RR 1.10, 95% CI 1.06–1.14) and preeclampsia (RR 1.11, 95% CI 1.07–1.16). No associations were observed for gestational diabetes.

**Table 3:** Associations between occupational noise exposure in relation to hypertensive disorders of pregnancy (HDP), preeclampsia, and gestational diabetes. [RR=relative risk; CI=confidence interval]

Noise exposure [dB(A)]	Exposed cases	Crude (N=1 109 516)		Adjusted a(N=1 087 944)	
		
	(Crude/adjusted)	RR	95%CI	RR	95%CI
HDP					
<70	28 252/27 714	1.00		1.00	
70–74	8603/8402	1.01	0.98–1.03	1.02	0.99–1.05
75–80	2382/2328	0.98	0.94–1.03	1.00	0.95–1.05
80–85	5408/5313	1.04	1.01–1.08	1.10	1.06–1.14
≥85	421/410	1.09	0.98–1.20	1.03	0.93–1.14
Preeclampsia					
<70	21 330/20 921	1.00		1.00	
70–74	6631/6478	1.03	1.00–1.06	1.02	0.98–1.05
75–80	1866/1820	1.02	0.97–1.07	1.00	0.94–1.06
80–85	4213/4140	1.08	1.04–1.11	1.11	1.07–1.16
≥ 85	340/332	1.16	1.04–1.30	1.06	0.94–1.19
Gestational diabetes					
<70	6220/6092	1.00		1.00	
70–74	2019/1959	1.08	1.02–1.13	0.99	0.93–1.05
75–80	566/543	1.06	0.97–1.16	0.93	0.84–1.04
80–85	1213/1178	1.06	1.00–1.13	0.98	0.90–1.06
≥85	103/98	1.20	0.99–1.46	0.93	0.75–1.16

a Adjusted for: age, smoking, education, country of birth, parity, employment status, particles, physical load, job strain, and exposure to low temperatures.

We conducted a separate analysis restricting to women who were in their first pregnancy and working full-time, the part of the full sample who were more likely to be present at work and thus more likely to be exposed to the levels within the assigned noise category. This resulted in a subsample of 349 300 pregnancies; results are presented in table 4. The same patterns were seen as in table 3, but with slightly stronger associations. The second highest noise category showed a statistically significant increased association of 1.12 (95% CI 1.05–1.18) for HDP and 1.14 (95% CI 1.07–1.22) for preeclampsia. For the highest noise category, the RR were increased, but not statistically significant (RR 1.06, 95% CI 0.92–1.22 for HDP and RR 1.09, 95% CI 0.93–1.28 for preeclampsia). Results for gestational diabetes did not follow the same patterns as table 3. For this sample, we observed that both 80–85 dB(A) (RR 1.23, 95% CI 1.04–1.46) and the ≥85 dB(A) (RR 1.57, 95% CI 1.10–2.24) categories were associated with increased risks when compared to those unexposed to noise. Analyses investigating an interaction between noise and age did not show evidence of any multiplicative interaction (data not shown).

**Table 4:** Associations between occupational noise exposure in relation to hypertensive disorders of pregnancy (HDP), preeclampsia, and gestational diabetes for first-time pregnancies with full-time employment. [RR=relative risk; CI=confidence interval]

Noise exposure [dB(A)]	Exposed cases	Crude (N=349 300)		Adjusted a (N=341 673)	
		
	(Crude/ adjusted)	RR	95%CI	RR	95%CI
HDP					
<70	12 788/12 565	1.00		1.00	
70–74	3561/3493	1.03	0.99–1.06	1.03	0.99–1.08
75–80	1140/1124	1.00	0.94–1.06	1.02	0.95–1.09
80–85	2306/2270	1.09	1.04–1.13	1.12	1.05–1.18
≥85	215/212	1.08	0.95–1.23	1.06	0.92–1.22
Preeclampsia					
<70	9954/9786	1.00		1.00	
70–74	2807/2759	1.04	1.00–1.08	1.03	0.98–1.08
75–80	922/907	1.04	0.97–1.11	1.01	0.93–1.10
80–85	1873/1848	1.13	1.08–1.19	1.14	1.07–1.22
≥85	181/179	1.17	1.02–1.35	1.09	0.93–1.28
Gestational diabetes					
<70	1537/1510	1.00		1.00	
70–74	460/448	1.10	0.99–1.22	1.11	0.98–1.26
75–80	137/133	0.99	0.83–1.18	0.93	0.75–1.15
80–85	291/283	1.14	1.01–1.29	1.23	1.04–1.46
≥ 85	40/39	1.68	1.23–2.29	1.57	1.10–2.24

a Adjusted for: age, smoking, education, country of birth, particles, physical load, job strain, and exposure to low temperatures

## Discussion

In this nationwide cohort study, exposure to noise at the workplace was associated with an increase in HDP and preeclampsia, notably for first-time pregnant women who worked full-time, the subsample of the full cohort we believe are more likely to be truly exposed. These associations were present beginning at 80 dB(A); however, were not statistically significant at levels >85 dB(A). Occupational noise exposure was further associated with an increase in gestational diabetes among first-time pregnant women working full-time who were exposed to noise levels >80 dB(A).

Previous studies have found conflicting results regarding noise exposure and pregnancy outcomes. One environmental study reported an increased risk for severe and early onset preeclampsia but not for mild and late onset (29). Three occupational studies find some increased, but nonsignificant, risk of gestational hypertension and/or preeclampsia associated with noise exposure in the workplace (16, 17, 19), while two studies (18, 20) find no associations. Even among studies reporting an increased risk, results are inconsistent. One of these, found a significantly increased association only among women who are also exposed to shift work (16), while another found a nonsignificant increase among parous women only (17). Risks obtained from this study fall somewhere between those reported previously. All these studies, however, are small and adjust for a limited number of pregnancy, lifestyle, and demographic confounders. Additionally, the few that adjust for other working conditions only adjust for shift work or physical load. To our knowledge, this is the first study to account for several job exposures, which lessens the likelihood that our results are due to other job exposures. There are no previous studies that investigate occupational noise and gestational diabetes. However, one study investigating environmental noise report odds ratios in the highest quartile of nighttime noise that are similar to our findings (30).

Our results for the 80–85 dB(A) category were largely driven by the childcare workers, which make up the majority of this group. Because data for this study come from a birth register with routine data collection, we do not believe that women who work in childcare and have a pregnancy outcome are disproportionately represented in the dataset. Restricting the sample and removing child care workers gives the similar results as in the full sample, except for the sub-group of restaurant and kitchen workers, whose inclusion did not show an increased risk (data not shown). Further studies should be conducted to elucidate these findings.

The subsample analysis of women who were in their first pregnancy and working full-time showed a similar effect for both HDP and preeclampsia. Preeclampsia is generally considered to be a disorder of the first pregnancy (3). In our sample, even though women who were in first pregnancies and worked full-time accounted for approximately one third of the full sample, almost half of the HDP and preeclampsia cases were in this group. Therefore, it is unsurprising that we see similar results in the general cohort.

While we observed no increased risk of gestational diabetes in association with noise exposure in the full cohort, we observed strong risks among the subsample of first pregnancies. Gestational diabetes recurrence is known to be high among those who had it during their first pregnancy, and, therefore, it might seem counterintuitive that risks are not seen in the full cohort including both first and successive pregnancies (31). We speculate that there is a lower likelihood of misclassification in the subsample of first pregnancies who work full-time, since it is possible that women who had a previous complication may change their tasks, go on leave, work less than full-time, or request to be reassigned. These women may be more absent or working less due to childcare responsibilities. Thus, even though results have lower precision due to the reduced sample size, estimates are more likely to be accurate because this subsample is more likely to be exposed to the assigned noise levels.

To date, the mechanism through which a woman develops HDP and gestational diabetes is unclear. For cardiovascular and metabolic diseases in general, noise is thought to induce a stress response, activating the sympathetic nervous and endocrine systems, leading to increased blood pressure, vascular damage, and metabolic disorders through oxidative stress and increased inflammation (10). Because pregnancy itself is characterized by increased hypothalamus-pituitary-adrenal axis function and concentration of stress hormones (13), the same pathway via which noise can lead to cardiovascular and metabolic disease. Therefore, it is plausible that the added stress response due to noise exposure can influence a woman’s likelihood of developing these pregnancy disorders.

With this mechanism in mind, one would expect to find the greatest risk at levels >85 dB(A), in other words, increasing levels of noise leading to an increased stress response; however, in our data this was not the case. One explanation could be that at these levels, workers are required to use hearing protection, which would protect mothers from the any adverse effects of noise. At the 80–85 dB(A) levels, workers are required to have access to, but do not necessarily have to use, hearing protection. One study, however, found no decrease in the risk of gestational hypertension among women who self-reported use of hearing protection (16). Regardless, the effect of hearing protection on this association is an area that warrants further exploration, not in the least because it may offer effective mitigation strategies. Another explanation is that perhaps women who are exposed to these high levels of noise are more likely to have jobs that would require them to be reassigned or go on leave, and in this case, women in the highest categories would not be exposed for the entirety of the pregnancy. Lastly, occupations with the highest noise level are also exposed to many other factors, and though we have checked for multicollinearity, it is possible that the inclusion of a combination of occupational factors attenuated the results.

This study has some limitations. Regarding exposure ascertainment, the noise levels provided by the JEM are not measured on an individual level, and thus misclassification can occur. The JEM were developed for general use based on measurements on both male and female workers and expert knowledge, without consideration for any particular outcome. Therefore, any misclassification is most likely non-differential and, as such, would only attenuate the risk reported. We did not have information on use of hearing protection. As mentioned, it is possible that the lower risk and lack of significant findings in the highest exposure category is due to use of hearing protection, but we could not test this hypothesis. Similarly, we did not have information on task reassignment. We did have information on pregnancy leave; however, because diagnoses were only given at the end of the pregnancy, we had no way to ascertain that the leave preceded any issues arising from HDP or gestational diabetes. Thus, we could not account for duration of exposure in our analyses. Due to local regulations, task reassignment and leave would most often be associated with physically strenuous work, for which our results are adjusted. Finally, we did not have information on the percentage of time part-time employees work, which can also affect the extent of exposure.

On the other hand, this study benefitted from a large sample and a prospective design. Even though HDP and gestational diabetes are the most common conditions of pregnancy, they are still relatively rare in the population. At the same time, the multifactorial nature of these disorders indicates there is likely no one major causative factor. Therefore, only a nationwide register this large can capture the number of exposed cases needed to detect the magnitude of differences expected. Additionally, because the data covers approximately 99% of all births in Sweden, results are generalizable to all pregnant women in Sweden. Another advantage is that data from this national, well-established register were collected as part of routine maternal care by healthcare professionals with nearly complete coverage of all pregnancies; therefore, data are more likely to be accurate and consistent for the entire sample. In this study, we were also able to control for several other exposures that confound the association between noise and pregnancy disorders via other JEM. These JEM were developed by experts in their respective fields and are based on the Swedish workforce, which should reduce misclassification at the occupational groups level.

In conclusion, this study indicates that there is an increased risk in HDP associated with exposure to noise at levels >80 dB(A). There is also an increased risk for gestational diabetes associated with these levels for first-time pregnant women who work full-time. Additional studies are needed to confirm this association, as well as investigate whether hearing protection can mitigate these risks.
